# Immediate Effects of Anodal Transcranial Direct Current Stimulation on Postural Stability Using Computerized Dynamic Posturography in People With Chronic Post-stroke Hemiparesis

**DOI:** 10.3389/fnhum.2020.00341

**Published:** 2020-08-27

**Authors:** Jing Nong Liang, Leonard Ubalde, Jordon Jacklin, Peyton Hobson, Sara Wright-Avila, Yun-Ju Lee

**Affiliations:** ^1^Department of Physical Therapy, University of Nevada, Las Vegas, Las Vegas, NV, United States; ^2^Interdisciplinary Doctoral Program in Neuroscience, University of Nevada, Las Vegas, Las Vegas, NV, United States; ^3^Department of Industrial Engineering and Engineering Management, National Tsing Hua University, Hsinchu, Taiwan

**Keywords:** transcranial direct current stimulation, post-stroke hemiparesis, postural control, dynamic posturography, center of gravity, fear of falling

## Abstract

Postural stability is commonly decreased in individuals with chronic post-stroke hemiparesis due to multisystemic deficits. Transcranial direct current stimulation (tDCS) is a non-invasive method to modulate cortical excitability, inducing neuroplastic changes to the targeted cortical areas and has been suggested to potentially improve motor functions in individuals with neurological impairments. The purpose of this double-blinded, sham-controlled study was to examine the acute effects of anodal tDCS over the lesioned motor cortex leg area with concurrent limits of stability training on postural control in individuals with chronic post-stroke hemiparesis. Ten individuals with chronic post-stroke hemiparesis received either anodal or sham tDCS stimulation over the lesioned leg region of the motor cortex while undergoing 20 min of postural training. The type of stimulation to receive during the first session was pseudorandomized, and the two sessions were separated by 14 days. Before and immediately after 20 min of tDCS, the 10 m walk test, the Berg Balance Scale, and dynamic posturography assessments were performed. After a single session of anodal tDCS with concurrent postural training, we observed no changes in clinical measures of balance and walking, assessed using the Berg Balance Scale and 10 m walk test. For dynamic posturography assessments, participants demonstrated improvements in adaptation responses to toes-up and toes-down perturbations, regardless of the type of tDCS received. Additionally, improved performance in the shifting center of gravity was observed during anodal tDCS. Taken together, these preliminary findings suggest that tDCS can potentially be used as a feasible approach be incorporated into the rehabilitation of chronic post-stroke individuals with issues related to postural control and fear of falling, and that multiple sessions of tDCS stimulation may be needed to improve functional measures of postural control and walking.

## Introduction

Individuals with post-stroke hemiparesis are at higher risks for falls, which can be debilitating, negatively impacts the quality of life, and poses a significant burden on health care costs (Sattin, 1992). Falls can involve multiple factors, including-individual related intrinsic factors, environment-related extrinsic factors, and activity-related behavioral factors (Tinetti et al., 1988; Nevitt et al., 1989). Postural instability has been identified as a major intrinsic risk factor for falls (Tinetti et al., 1988; Shumway-Cook et al., 1997). Previous evidence demonstrated the involvement of the leg motor area in postural tasks, static standing, responding to perturbations greater than postural sway during static standing, and locomotion (Beck et al., 2007; Tokuno et al., 2009). Furthermore, functional asymmetry exists between two motor areas in the selection of appropriate postural strategies (Cioncoloni et al., 2016). After lesions to the cortex, such as in a stroke, postural stability is commonly decreased as a result of deficits in the sensory, musculoskeletal, perceptual, and cognitive systems (Duncan, 1994).

Transcranial direct current stimulation (tDCS) is a non-invasive method that can be used to modulate cortical excitability by applying a direct weak electric current to the brain (Nitsche and Paulus, 2000). The modulatory effect depends on the positioning and polarity of the electrodes on the scalp; anodal stimulation results in increased cortical excitability, and cathodal stimulation results in decreased cortical excitability (Nitsche et al., 2005, 2007). This type of non-invasive brain stimulation is relatively inexpensive when compared with repetitive transcranial magnetic stimulation and epidural stimulation, and thus widely used in studies examining neuromodulation (Nitsche and Paulus, 2011). Due to its close distance from the skull surface and small variability in orientation inter-individually, studies on the effects of tDCS delivered to the upper limb area of the primary motor cortex to modulate cortical excitability (Nitsche and Paulus, 2000; Edwards et al., 2009) and upper limb functions (Hummel et al., 2005, 2010) have been extensively reported. With respect to the lower extremities, because the leg motor area is located deeper and oriented more vertically relative to the skull, less literature is reported compared with that in the upper extremities. Anodal tDCS when applied to the leg areas of the primary motor cortex has been reported to result in increased excitability of the non-impaired corticospinal tracts of the tibialis anterior muscles as assessed using motor-evoked potentials (Jeffery et al., 2007), increased toe pinch force output in the non-neurologically impaired (Tanaka et al., 2009), and improved paretic knee extensor strength in individuals chronically post-stroke (Tanaka et al., 2011). With respect to balance and postural control, positive effects have been reported with low-frequency repetitive transcranial magnetic stimulation to improve static postural stability post-stroke (Forogh et al., 2017) but not with intermittent theta-burst stimulation (Lin et al., 2019), and epidural stimulation has been shown to improve static standing balance in individuals with chronic spinal cord injuries (Rejc et al., 2015). Furthermore, other non-invasive methods of neuromodulation, such as prismatic adaptation, have been shown to activate compensatory postural adjustments to achieve improved body stability in the non-neurologically impaired (Bonaventura et al., 2020). Few studies, however, have investigated the effects of tDCS delivered to the leg motor area on balance and postural control in non-neurologically impaired individuals (Craig and Doumas, 2017; Yosephi et al., 2018; Xiao et al., 2020) and even more limited in individuals post-stroke (Sohn et al., 2013).

The primary purpose of this study was to examine the immediate effects of anodal tDCS over the lesioned motor cortex in combination with limits of stability training on postural control in individuals with chronic post-stroke hemiparesis. We hypothesized that after 20 min of anodal tDCS stimulation over the lesioned leg motor area with concurrent limits of stability training, individuals with chronic post-stroke hemiparesis would show improvements in gait and balance, assessed using the 10 m walk test, Berg Balance Scale, and functional reach test, and postural control, assessed using dynamic posturography.

## Materials and Methods

### Participants

Ten individuals (age ± *SD* = 58.96 ± 9.56 years; four females and six males) who had sustained a single cortical or subcortical stroke at least 6 months before the study, and were able to walk independently without assistive devices, were recruited in this study (see Table 1 for participant characteristics). Participants were recruited via word of mouth and advertising flyers from stroke support groups in the local community. Exclusion criteria included a history of seizures, metallic implants, central nervous system lesions other than the stroke, and any orthopedic conditions in the lower extremities at the time of recruitment. A safety screening questionnaire was administered before recruitment to ensure safety (Charalambous et al., 2019). Before participation, each participant received written and verbal information about the experiment procedures before signing the informed consent. The Institutional Review Board at the University of Nevada, Las Vegas, approved the protocol (protocol #:1330419).

**TABLE 1:** Participant characteristics (*N* = 10).

Participant ID	Paretic limb (L/R)	Time post-stroke (years)	LE Fugl-Meyer motor function score (/34)
01	L	7.55	18
02	L	19.09	21
03	R	1.98	19
04	R	4.10	21
05	R	3.38	32
06	R	2.32	24
07	R	3.84	26
08	L	10.00	30
09	R	6.42	32
10	L	5.67	29

Mean	4L/6R	6.43	25.20
SD		5.09	5.35

### Procedures

This was a double-blinded, sham-controlled, crossover study. Each participant attended two sessions separated by at least 14 days. For each session, the participant received either anodal or sham stimulation over the leg area of the lesioned motor cortex. The type of stimulation to be received was randomized in session one for participant 1 using a coin toss. After randomizing the stimulation type for the first participant, each participant after that received the other type of stimulation that was not administered in the first session of the previous participant. In their second session, participants received the other type of stimulation that was not administered in their first session. Throughout the study, the same researcher was responsible for administering tDCS stimulation. The other researchers, blinded to the stimulation type, assessed the outcome measures. All data collection sessions were scheduled on weekday afternoons between 12 and 5 p.m. The two sessions for each participant were kept at the same time of the day. Participant recruitment and data collection ran from November 2018 to May 2019.

### Transcranial Direct Current Stimulation

A direct current stimulator (NeuroConn DC-Stimulator PLUS, Germany) delivered direct current via two conductive rubber electrodes placed in saline-soaked sponges (5 × 5 cm each). Using the 10:20 EEG system, the anode was placed 1 cm anterior to the cranial vertex, and the cathode was placed over the supraorbital area (Klem et al., 1999). Anodal tDCS was applied over the lesioned primary motor cortex using a 2 mA current with a ramp-up period of 30 s, stimulation period at 2 mA for 19 min, and ramp-down period to 0 mA in the final 30 s, for a total duration of 20 min (Yosephi et al., 2018). Sham tDCS followed a similar protocol and arrangement but stimulation at 2 mA for 30 s, after which the current was ramped-down and turned off for the rest of the treatment. This procedure blinded participants to the type of stimulation they received while preventing any changes in cortical excitability (Nitsche et al., 2003, 2008).

During the 20 min of tDCS application, participants stood on a force platform (Bertec Corporation, Columbus, OH), with their center of the center of gravity (CoG) represented on a screen in front of them. They were asked to shift their weight in eight different directions (forward, backward, right, left, forward-right, forward-left, backward-right, and backward-left) as quickly and accurately, without changing their feet positions, so that their CoG coincides the presented target on the screen. The CoG traces during the 20 min intervention were recorded and their performance variables computed, including reaction time, movement velocity, and endpoint excursion. Movement velocity is the average speed of CoG movement from the individual’s center to each of the eight targets, expressed as degrees/second. Endpoint excursion measures the limits of self-initiated movements as the individual shifts his/her CoG toward the theoretical limit in each of the eight target directions without loss of balance, expressed as a percentage of limits of stability. For reaction time, a smaller value indicates better performance, whereas, for all other variables, a greater value indicates better limits of stability. During the 20 min of tDCS application and CoG shift training, participants were not provided with any support to hold on with their hands. Participants wore a safety harness secured to an overhead frame. The harness was adjusted such that it did not provide any support to the participants during standing but acted as a safety measure that would catch them in case they lose their balance.

### Assessment of Gait and Postural Control

**FIGURE 1:**
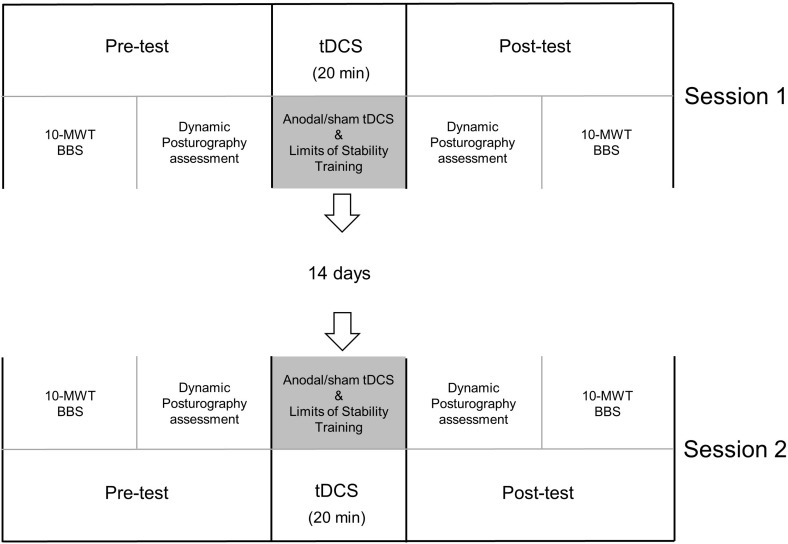
Timeline. Each participant attended two sessions separated by at least 14 days. For each session, the participant received either anodal or sham stimulation for 20 min and concurrent limits of stability training. Before and immediately after tDCS, clinical assessments [10 m walk test (10-MWT) and Berg Balance Scale (BBS)] and dynamic posturography assessments were performed.

Before and immediately after the 20 min of tDCS application, a researcher who was blinded to the type of stimulation the participant received would evaluate the participant’s performance using each of the following: Berg Balance Scale, Forward Reach Test, 10 m walk test, and the adaptation test and motor control test using the Bertec Advantage Computerized Dynamic Posturography system (Bertec Corporation, Columbus, OH) (Figure 1).

The Berg Balance Scale is a 14-item scale used to assess balance during static and dynamic tasks of varying difficulties, and has been used to assess fall risks (Berg et al., 1992; Bogle Thorbahn and Newton, 1996; Blum and Korner-Bitensky, 2008). Participants were instructed to perform 14 varying, functional tasks such as sit-to-stand, forward reach, and balancing on one foot, with each task being graded from 0 to 4 based on the quality of movement and time to complete each task.

Along with the total score of the Berg Balance Scale, the scores of the Forward Reach Test were also examined independently, the performance of which can predict fall risks (Behrman et al., 2002). Participants were instructed to stand next to a yardstick mounted at shoulder level on a wall. Participants were then asked to remain upright while elevating both upper extremities to shoulder level and form a fist with both hands. The starting position, which was determined by the starting distance of the third metacarpophalangeal joint in reference to the yardstick, was then recorded by the researcher performing the assessment. The researcher then instructed the participant to reach forward as far as possible without losing their balance or taking a step forward. As the participant reached forward, the researcher would record the farthest distance reached by the third metacarpophalangeal joint. The difference between the two measurements was calculated to obtain the distance for the Forward Reach Test.

In the 10 m walk test, participants were instructed to walk in a straight line for 10 m, and the time taken to complete was recorded with a stopwatch. The test was performed in two conditions: a dynamic start condition and a static start condition (Scivoletto et al., 2011). In the dynamic start condition, the participant walked for 4 m before the researcher started timing for the next 10 m. The static start condition had the participant immediately start the 10 m walk from a static standing position. A 1 min break was provided between each condition, as per protocols in previous studies (Scivoletto et al., 2011).

In the adaptation test, the participant was instructed to stand on the force platform and maintain balance with minimal sway, when the platform generated a toes up or toes down perturbation. The amount of anterior–posterior sway to overcome the postural instability (sway energy) was measured.

In the motor control test, the participant was instructed to stand on the force platform, and an expected translation of the platform would be generated in graded magnitudes. Each participant was perturbed with three trials of each magnitude of forward and backward translations (small, medium, and large). The time to recover from perturbations (latency between translation onset and force response) was recorded for each trial.

### Statistical Analysis

A 2 (stimulation: anodal, sham) × 2 (time: pre, post) repeated measures ANOVA was conducted on each dependent variable. Where there was a significant interaction effect, we further examined simple main effects using a repeated measures ANOVA with Bonferroni correction. Paired samples *T*-tests were conducted on each CoG performance variable computed from the limits of stability training during the intervention. Significant main effects were reported if there were no significant interactions. *P*-values less than or equal to 0.05 were considered statistically significant.

## Results

### Clinical Assessments

For the Berg Balance Scale, the two-way ANOVA revealed no significant interaction between stimulation and time [*F*(1, 9) = 0.053, *p* = 0.823]. Furthermore, no significant differences were found for the main effects of stimulation [*F*(1, 9) = 0.363, *p* = 0.562] and time [*F*(1, 9) = 3.655, *p* = 0.088]. This suggests that the type of stimulation did not cause a change in scores after the intervention and over time (Table 2).

**TABLE 2:** Comparison of clinical assessment measures (mean ± SD) pre and post anodal and sham tDCS stimulation.

		Anodal	Sham
Berg Balance Scale (/56)	Pre	52.70 ± 4.16	52.30 ± 4.99
	Post	53.70 ± 3.53	53.20 ± 4.39
Forward reach (in)	Pre	8.52 ± 2.99	9.12 ± 3.16
	Post	8.98 ± 2.89	9.56 ± 2.49
10 m walk with dynamic start (m/s)	Pre	1.09 ± 0.36	1.05 ± 0.36
	Post	1.13 ± 0.37	1.06 ± 0.33
10 m walk with static start (m/s)	Pre	0.97 ± 0.29	0.97 ± 0.30
	Post	1.01 ± 0.29	0.97 ± 0.30

For the Forward Reach Test, two-way ANOVA revealed no significant interaction between stimulation and time [*F*(1, 9) = 0.00, *p* = 0.985]. Furthermore, no significant differences were found for the main effects of stimulation [*F*(1, 9) = 0.956, *p* = 0.354] and time [*F*(1, 9) = 0.705, *p* = 0.423]. This suggests that the type of stimulation did not cause a change in forward reach distance after the intervention and over time (Table 2).

For the 10 m walk test with a dynamic start, two-way ANOVA revealed no significant interaction between stimulation and time [*F*(1, 9) = 0.348, *p* = 0.57]. Furthermore, no significant differences were found for the main effects of stimulation [*F*(1, 9) = 2.517, *p* = 0.147] and time [*F*(1, 9) = 3.885, *p* = 0.080] (Table 2). Similarly, for the 10 m walk test with a static start, two-way ANOVA revealed no significant interaction between stimulation and time [*F*(1, 9) = 2.275, *p* = 0.166]. Furthermore, no significant differences were found for the main effects of stimulation [*F*(1, 9) = 0.846, *p* = 0.382] and time [*F*(1, 9) = 1.071, *p* = 0.328] (Table 2).

### Dynamic Posturography Assessments

For the adaptation test, in the toes up perturbation condition, there was no significant interaction between stimulation and time [*F*(1, 9) = 0.314, *p* = 0.589]. No significant differences were found for the main effects of stimulation [*F*(1, 9) = 0.279, *p* = 0.61]. There was a statistically significant main effect of time [*F*(1, 9) = 30.55, *p* < 0.001], where a reduction in the amount of anterior–posterior sway to overcome the postural instability induced by the toes up perturbation after the intervention (Pre = 80.54 ± 17.10, Post = 65.84 ± 11.48) was observed, suggesting that, on average, all participants responded to the toes up perturbation with less anterior–posterior sway after the intervention compared with before the intervention, regardless of the type of stimulation received. Similarly, in the toes down perturbation condition, there was no significant interaction between stimulation and time [*F*(1, 9) = 1.826, *p* = 0.21]. No significant differences were found for the main effects of stimulation [*F*(1, 9) = 2.009, *p* = 0.190]. There was a statistically significant main effect of time [*F*(1, 9) = 8.271, *p* = 0.018], where a reduction in the amount of anterior–posterior sway to overcome the postural instability induced by the toes down perturbation after the intervention (Pre = 75.33 ± 11.96; post = 67.21 ± 9.58) was observed, suggesting that, on average, all participants responded to the toes down perturbation with less anterior–posterior sway after the intervention compared with before the intervention, regardless of the type of stimulation received (Table 3).

**TABLE 3:** Comparison of dynamic posturography assessments (mean ± SD) pre and post anodal and sham tDCS stimulation.

	Perturbation			Anodal	Sham	Averaged across stimulations
Adaptation test	Toes up	Sway energy (mm/s)	Pre	80.94 ± 13.98	80.14 ± 20.53	80.54 ± 17.10
			Post	67.26 ± 11.34	64.42 ± 12.04	65.84 ± 11.48^‡^
	Toes down	Sway energy (mm/s)	Pre	78.48 ± 10.87	72.18 ± 12.72	75.33 ± 11.96
			Post	68.48 ± 10.01	65.94 ± 9.48	67.21 ± 9.58^‡^

Motor control test	Forward translation	Latency (ms)	Pre	139.60 ± 9.40	135.70 ± 13.50	137.65 ± 11.49
			Post	138.00 ± 8.76	134.40 ± 15.02	136.20 ± 12.11
	Backward translation	Latency (ms)	Pre	139.60 ± 9.40	135.70 ± 13.50	137.65 ± 11.49
			Post	138.00 ± 8.76	134.70 ± 14.86	136.35 ± 11.99

*^‡^Indicates statistically significant difference from Pre-test (p ≤ 0.05) when averaged across stimulation types.*

In the motor control test, in the forward translation perturbation condition, there was no significant interaction between stimulation and time [*F*(1, 9) = 0.034, *p* = 0.858]. No significant differences were found for the main effects of stimulation [*F*(1, 9) = 0.961, *p* = 0.353] and time [*F*(1, 9) = 0.614, *p* = 0.453]. Similarly, in the backward translation perturbation condition, there was no significant interaction between stimulation and time [*F*(1, 9) = 0.163, *p* = 0.696. No significant differences were found for the main effects of stimulation [*F*(1, 9) = 0.871, *p* = 0.375] and time [*F*(1, 9) = 0.498, *p* = 0.498] (Table 3).

### Limits of Stability Training During Transcranial Direct Current Stimulation

For CoG performance variables computed from the limits of stability training, paired-samples *T*-tests revealed greater movement velocity in the backward direction during anodal stimulation (3.56°/s ± 1.94) compared with sham (1.85°/s ± 1.35) (*p* = 0.025), and greater backward endpoint excursion during anodal stimulation (72.53% ± 34.86) compared with sham (36.71% ± 33.84) (*p* = 0.026) only.

## Discussion

Contrary to our hypothesis, our results showed that a single session of anodal tDCS with limits of stability training was not effective in improving clinical measures, specifically, Berg Balance Scale scores, Forward Reach Test, and overground walking speed. Additionally, we did not observe any improvements with anodal tDCS in the adaptation test and motor control test measures as assessed using dynamic posturography. However, we observed an improvement in the ability of the participants to move their CoG to their stability limits without losing their balance during the application of anodal stimulation.

Immediately after a single session of anodal tDCS application together with limits of stability training, we did not observe improvements in clinical assessments, specifically the Berg Balance Scale, Forward Reach Test, and overground walking speed. Clinical assessments, although relatively quick and easy to administer and did not require expensive equipment, are, however, subjective and not sufficiently responsive to capture small changes in the ability to control balance (Blum and Korner-Bitensky, 2008). Furthermore, Berg Balance Scale has been identified to be a good predictor of fall status in non-neurologically impaired elderly, where individuals scoring high on the Berg Balance Scale have relatively low fall risks and individuals scoring less than 40 have a high probability for falls (Shumway-Cook et al., 1997). The participants recruited in this study, despite the stroke lesion, scored a mean of 52 on the Berg Balance Scale, suggesting that they were of a relatively higher function for balance control. Thus, the results might not be extrapolated to individuals post-stroke with lower balance function. Furthermore, examining kinetics and kinematics during balance assessments could overcome this ceiling effect and provide more quantitative insight into participant performance.

Effectiveness of anodal tDCS applied to the primary motor cortex on improving static posture and balance and gait parameters have previously been reported in children with cerebral palsy and Parkinson’s disease and an individual with hemiparesis (Duarte Nde et al., 2014; Kaski et al., 2014; Dumont et al., 2015). With respect to dynamic postural control as assessed using dynamic posturography, due to the lack of comparable studies in the existing literature, we are only able to compare our current observations with previous findings examining the effects of anodal tDCS on static postural stability in individuals with various postural deficits. In contrast to our findings, six sessions of anodal tDCS with postural training over 2 weeks improved Berg Balance Scale scores and static postural control in non-neurologically impaired individuals (Yosephi et al., 2018), and 10 sessions of anodal tDCS over 2 weeks improved static gait and functional performance in children with cerebral palsy (Duarte Nde et al., 2014). Together, these suggest that multiple sessions of stimulation may be needed to improve functional measures.

In this current study, we targeted the leg area of the lesioned primary motor cortex for anodal tDCS application. Post-stroke, postural control is likely more sensory-driven than anticipatory, as anticipatory mechanisms involve various cerebral regions, including cortical, subcortical, and subtentorial areas (Hocherman et al., 1988; Massion et al., 2004). A single session of anodal tDCS on the cerebellum improved balance and postural stability in older adults (Ehsani et al., 2017) but did not influence static postural control in young adults (Inukai et al., 2016), whereas cathodal tDCS over the cerebellum impaired static balance (Foerster et al., 2017). Additionally, with the immediate proximity of the supplementary motor area anatomically, placing the electrode over the leg motor area for tDCS stimulation could possibly result in effects in the supplementary motor area. Improvements in simple motor tasks such as visuomotor pinch force task, reaction times, accuracy, speed, and movement initiations have been previously reported with anodal tDCS over the supplementary motor area (Hayduk-Costa et al., 2013; Vollmann et al., 2013; Carlsen et al., 2015). Few have examined tDCS effects on complex motor behaviors associated with the lower extremities, including balance and posture (Kaski et al., 2013; Hupfeld et al., 2017). Interestingly, similar to our observed improvements in backward velocity in the limits of stability task, during anodal tDCS stimulation over the leg motor area, an earlier study observed improved balance speed during anodal tDCS application over the supplementary motor area (Hupfeld et al., 2017). Thus, anodal tDCS potentially improved the functional connectivity between the motor and supplementary motor areas underlying these observed improvements (Hamada et al., 2009). Future studies should systematically explore cortical areas optimally suited for tDCS stimulation to improve postural control. Due to the possibility of neural plasticity after stroke lesions chronically, these target cortical areas may be different than those in the non-neurologically impaired nervous system.

The improvements in sway energy observed in the adaptation tests immediately after the tDCS and postural training are likely from the 20 min of practice. Medium latency responses via the brainstem are likely responsible for these functional postural responses, in combination with the cerebellum, which adapts postural responses for optimality based on preceding experience (Jacobs and Horak, 2007). Although the impaired adaptation of the Ia afferent pathway via the spinal cord has been reported in tasks involving postural control and locomotion in people chronically post-stroke (Liang and Brown, 2015; Liang et al., 2019), they are likely not the main contributor to this observed improvement, as short-latency reflex pathway via the spinal cord is too weak to be functional for postural control.

**FIGURE 2:**
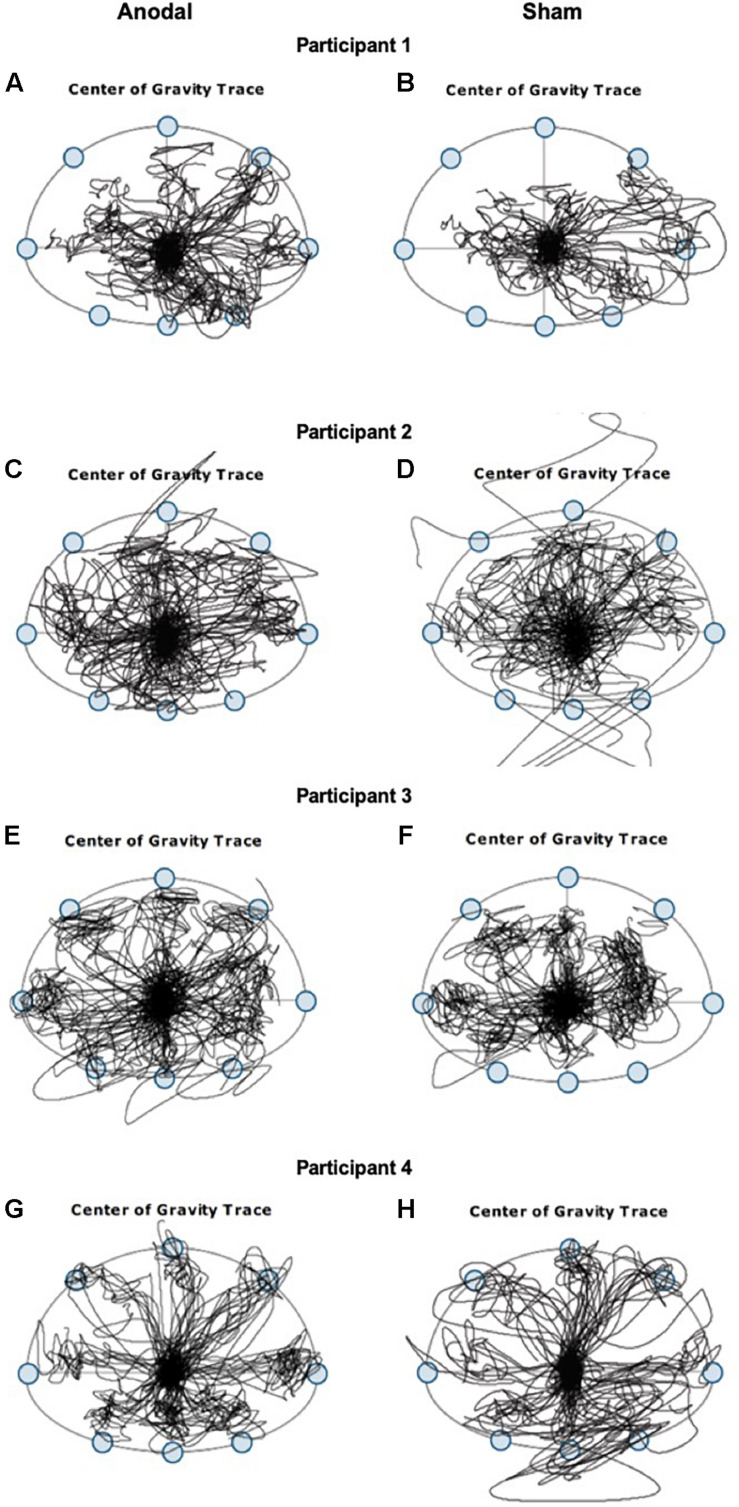
Center of gravity (CoG) traces from four representative participants with chronic post-stroke hemiparesis recorded during the 20 min of limits of stability training when tDCS stimulations were applied. Left panels **(A,C,E,G)** are CoG traces during anodal stimulation, and right panels **(B,D,F,H)** are CoG traces during sham stimulation.

Interestingly, despite the lack of improvement observed in assessments of dynamic postural control tasks immediately after anodal tDCS, we observed better performance in shifting the CoG toward stability limits in different directions without losing their balance during the application of anodal tDCS but not during sham (Figure 2). Stability limits are defined as the boundaries within which a body can maintain stability without changing the base of support (Shumway-Cook and Woollacott, 2012). In both participants 1 and 3, the area of CoG displacements covered during the limits of stability task is greater during the 20 min of anodal stimulation and smaller during the sham stimulation, in all directions. A smaller center of pressure excursions has been associated with older adults who are fearful of falling (Binda et al., 2003). Participants 2 and 4 lost their balance several times indicated by the CoG traces out of range during the sham stimulation but not during the anodal stimulation. In older adults, fear of falling contributed to scores mainly in the backward direction to measure limits of stability (Newton, 2001). Additionally, during anodal stimulation, we observed greater movement velocity and greater endpoint excursion in the backward direction but not during sham stimulation. The type of stimulation that each participant receives during the first session was pseudorandomized, and thus, the better performance observed is unlikely due to practice alone.

To the best of our knowledge, this is the first study to assess the acute effects of anodal tDCS and limits of stability training on improving postural control assessed using dynamic posturography in individuals with chronic post-stroke hemiparesis. Together, these preliminary observations suggest that anodal tDCS potentially can be used as a feasible approach for chronic post-stroke individuals with issues related to postural control and fear of falling. However, our small sample of 10 individuals with chronic post-stroke hemiparesis has limited us from examining the effects on different sites of lesion. Furthermore, this study was limited by convenience sampling, as all individuals recruited for the study were from the same stroke support group meeting held in the local community. Their past medical care and rehabilitation experience may have been relatively similar and thus may not be a true representation of the functional levels of individuals with chronic post-stroke hemiparesis. A future effort will extend these preliminary findings on a large scale to investigate the longer-term effects of tDCS on post-stroke postural control to reduce the risk of falls.

## Data Availability Statement

The raw data supporting the conclusions of this article will be made available by the authors, without undue reservation, to any qualified researcher.

## Ethics Statement

The studies involving human participants were reviewed and approved by the Institutional Review Board at the University of Nevada, Las Vegas. The participants provided their written informed consent to participate in this study.

## Author Contributions

JL, LU, JJ, PH, and SW-A: conceptualization, methodology, software, validation, formal analysis, investigation, resources, data curation, writing – original draft preparation, and funding acquisition. JL and Y-JL: writing – review and editing. JL: supervision. All authors contributed to the article and approved the submitted version.

## Conflict of Interest

The authors declare that the research was conducted in the absence of any commercial or financial relationships that could be construed as a potential conflict of interest.
